# Recurrent Selection for Transgene Activity Levels in Maize Results in Proxy Selection for a Native Gene with the Same Promoter

**DOI:** 10.1371/journal.pone.0148587

**Published:** 2016-02-19

**Authors:** Anastasia L. Bodnar, Megan N. Schroder, M. Paul Scott

**Affiliations:** 1 Iowa State University Interdepartmental Genetics Program, Ames, Iowa, United States of America; 2 United States Department of Agriculture, Agricultural Research Service, Corn Insects and Crop Genetics Research Unit, Ames, Iowa, United States of America; Institute of Genetics and Developmental Biology, Chinese Academy of Sciences, CHINA

## Abstract

High activity levels of a transgene can be very useful, making a transgene easier to evaluate for safety and efficacy. High activity levels can also increase the economic benefit of the production of high value proteins in transgenic plants. The goal of this research is to determine if recurrent selection for activity of a transgene will result in higher activity, and if selection for activity of a transgene controlled by a native promoter will also increase protein levels of the native gene with the same promoter. To accomplish this goal we used transgenic maize containing a construct encoding green fluorescent protein controlled by the promoter for the maize endosperm-specific 27kDa gamma zein seed storage protein. We carried out recurrent selection for fluorescence intensity in two breeding populations. After three generations of selection, both selected populations were significantly more fluorescent and had significantly higher levels of 27kDa gamma zein than the unselected control populations. These higher levels of the 27kDa gamma zein occurred independently of the presence of the transgene. The results show that recurrent selection can be used to increase activity of a transgene and that selection for a transgene controlled by a native promoter can increase protein levels of the native gene with the same promoter via proxy selection. Moreover, the increase in native gene protein level is maintained in the absence of the transgene, demonstrating that proxy selection can be used to produce non-transgenic plants with desired changes in gene expression.

## Introduction

The Illinois Long Term Selection Experiment is a well-known example of how recurrent selection can result in dramatic changes in phenotype, in this case protein or oil content. Starting with a single population in 1896, recurrent selection for and against grain protein content over 100 generations resulted in 32 and 4% protein respectively, compared to 8–12% protein in the starting population [1]. Recurrent selection for and against grain oil content resulted in 20 and 1% oil respectively compared to 4–6% oil in the starting population [1]. While the Illinois Long Term Selection Experiment is impressive in its longevity and in the large amount of divergence seen in the high and low lines while retaining genetic diversity, recurrent selection for a trait can also result in significantly different lines in just a few generations. For example, Scott et al. [2] used recurrent selection to change methionine content in maize: after 4 generations of selection, there was 17.6% difference between the high and low methionine lines. Other examples of successful recurrent selection programs in maize include increased prolificacy (number of ears) in the Golden Glow population [3], long and short ear length [4], and pseudostarchy endosperm or extreme sugary endosperm in a *sugary1* background [5].

It would be useful if the power of recurrent selection could be harnessed to increase transgenic protein production. High activity levels of a transgene can make a transgene easier to evaluate for safety and efficacy. High activity levels can also be important for production of transgenic protein, such as pharmaceutical or industrial proteins. Despite success with recurrent selection in improving non-transgenic traits, this method has only been reported as a method to increase transgene activity in one study. Hood et al. [6] used breeding to increase protein levels produced by a transgene with an embryo preferred promoter. They crossed transgenic lines to high-oil lines, followed by selection for high levels of the transgene-encoded protein. Here, we subject fluorescence of green fluorescent protein (GFP) controlled by the 27kDa gamma zein promoter to selection pressure with recurrent selection.

GFP is a convenient marker that produces fluorescence that is directly proportional to the amount of protein [7] and can be screened visually or quantified with fluorometry in whole seeds, requiring no processing to induce fluorescence. In maize, seed storage proteins called zeins make up about 50% of total protein in the endosperm [8] and are not expressed elsewhere in the plant [9]. Due to their localized activity at high levels, zein promoters are useful for many biotechnology applications [10], including production of proteins for extraction and for biofortification. The 27kDa gamma zein promoter was previously shown to drive high activity of GFP in maize endosperm [11].

Recurrent selection is only possible if the trait of interest can be easily quantified. Traits that are expensive or difficult to measure are unlikely to be subjected to recurrent selection, even if an increase in those traits would be useful. Thus it is not practical to alter the activity of most genes by recurrent selection. We propose that it may be possible to apply selection pressure to an easily quantifiable transgene with the same promoter as a native gene of interest in order to increase activity of that native gene.

This research investigates recurrent selection as a method to increase transgene activity and the effects of that selection on native genes. The primary hypothesis is that selection for high fluorescence of a 27kDa gamma zein GFP transgene fusion will result in higher activity of GFP in subsequent generations. The secondary hypothesis is that levels of the native 27kDa gamma zein will also increase due to selection pressure on the 27kDa gamma zein promoter, in a phenomenon we propose to call proxy selection.

## Materials and Methods

### Transgenic seed development

Maize seeds expressing GFP were developed and backcrossed to B73 for 3 generations by Shepherd [11]. Event P230-71-1 was selected because it expressed GFP well and appeared to be a single-copy integration event. The construct contained the *Zea mays* 27kDa gamma zein endosperm-specific promoter cloned from inbred Va26 (Genbank accession EF061093), the modified green fluorescent protein (GFP) gene sGFPs65T (Genbank accession ABB59985) [12], and the nos terminator sequence (modified from Genbank accession V00087). GFP and the nos terminator were from the pAct1IsGFP-1 plasmid [13].

### Development of segregating populations and seed production

**Fig 1:**
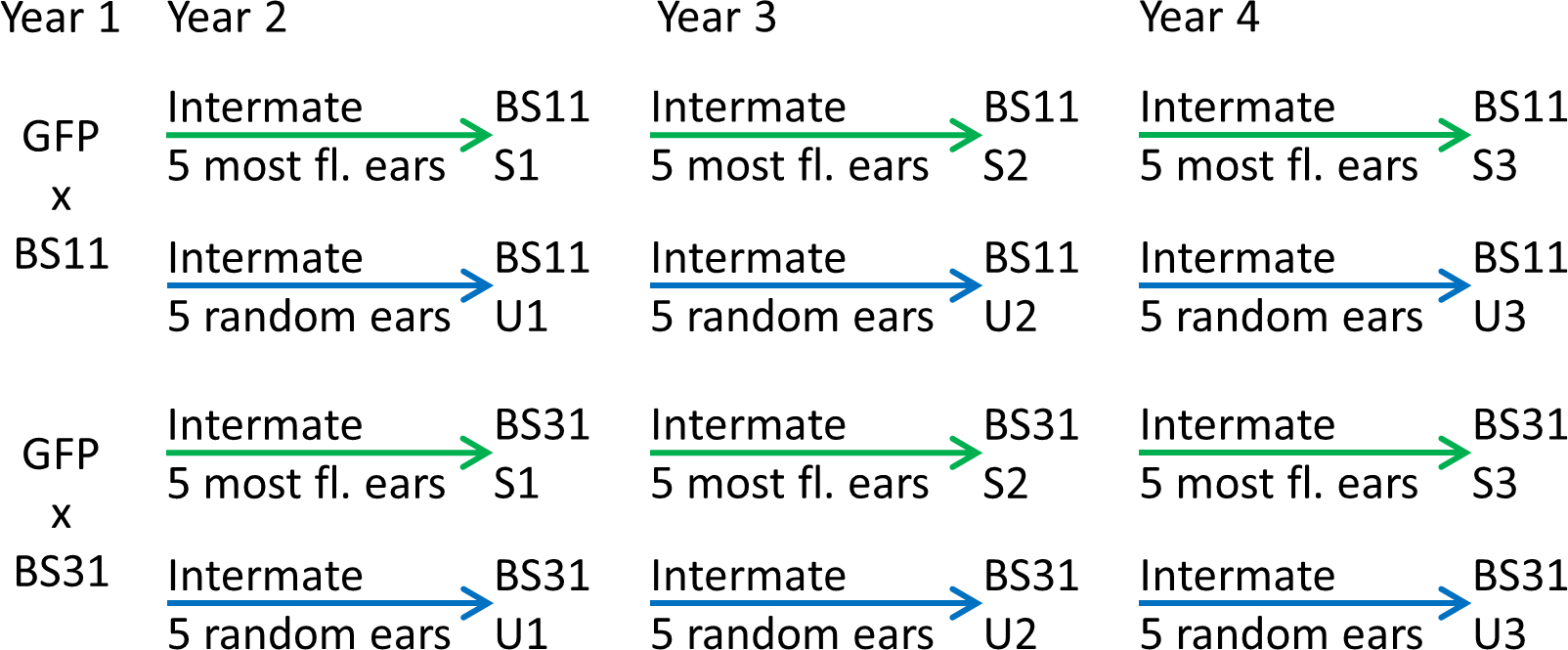
Breeding strategy used to develop selected and unselected populations over three generations. For the selected populations, the most fluorescent ears were the parents for the next generation. For the unselected populations, random ears were the parents for the next generation. The abbreviation fl. is for fluorescence.

The breeding plan is in Fig 1. All plants were grown at the Iowa State University Transgenic Farm in Ames, IA as follows: Year 1 in 2006, Year 2 in 2007, Year 3 in 2009, and Year 4 in 2010. There was no planting in 2008 due to field flooding. In year 1, the transgene was bred into two broad-based synthetic breeding populations: BS11 (derived from the Pioneer two-ear composite [14]) and BS31 (derived from FS8B [15]). This was done by crossing the homozygous transgenic inbred line with about 50 individuals of each breeding population. We tested seeds from the resulting full-sib ears for fluorescence as described below. These breeding populations provided the genetic variability needed for selection. Using two different breeding populations allowed us to determine if the two populations reacted similarly to selection or if any observed effects were specific to a single population. We harvested approximately 50 ears from each population each year and evaluated those ears for GFP fluorescence. To avoid selecting for homozygosity at the transgene locus in the selected populations, we used only ears that were visually segregating for visible GFP fluorescence to advance all populations. We compared selected populations to control populations that were random mated without selection.

In year 1, 50 randomly chosen seeds from each of the five ears with the highest mean fluorescence level were bulked and planted and the resulting plants intermated to create two selected populations (S1), one derived from BS11 and the second derived from BS31. Methods for fluorescence measurement are described below. In addition, we bulked and planted 50 randomly chosen seeds from each of five randomly chosen ears and intermated the resulting plants to create two unselected (control) populations (U1), one derived from BS11 and the second derived from BS31. In year 3, we tested ears from each S1 population for fluorescence, and selected ears were intermated as in year 1 to create S2. The unselected populations were advanced as described for year 2 to create U2. In year 4, the selected and unselected populations were advanced as in year 3 to create S3 and U3.

### Evaluation of selected populations

Twelve populations (two starting populations x selected or unselected x three generations) were evaluated in single plots in one experiment in 2010. Selected and unselected populations were randomly assigned positions in adjacent plots. Plots consisted of four rows of 50 seeds each. Plants within each population were intermated by hand using chain sib pollinations to avoid pollen flow from neighboring populations. We harvested a total of 448 ears, and randomly chose ears from each harvested population for the experiments. For determination of fluorescence, 30 ears from each population were evaluated as described below, for a total of 14,400 measurements.

### GFP screening

**Fig 2:**
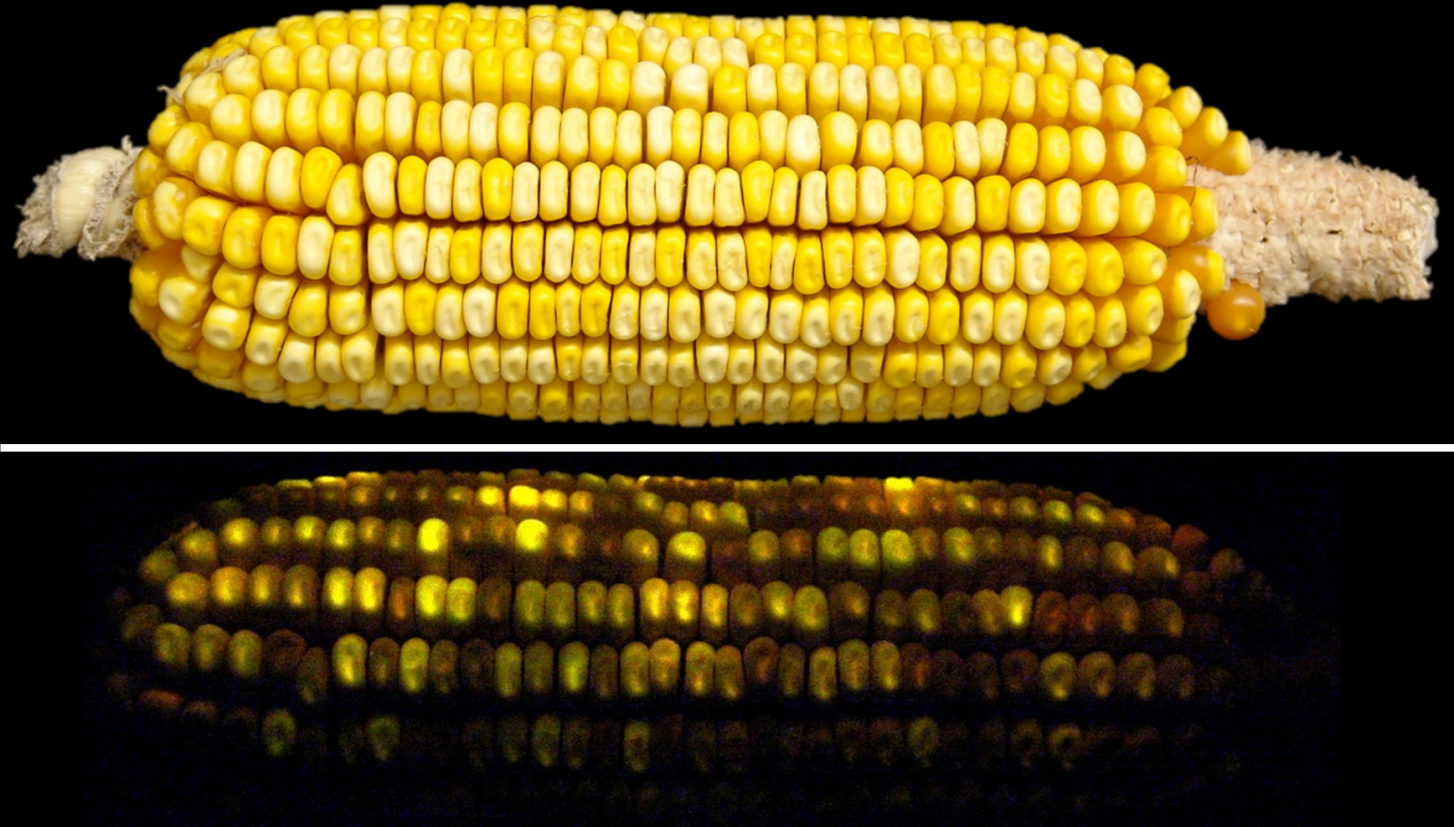
Maize segregating for various levels of GFP activity. The top panel is in white light and the bottom panel in blue light (485nm) with an orange filter (535nm).

A Dark Reader hand lamp (Clare Chemical, Dolores, CO) was used to visually screen seeds for GFP fluorescence. Quantification of fluorescence was conducted by measurement with a spectrofluorometer (Tecan, Mannedorf/Zurich, Switzerland) at 16 points within each well of a 6-well Costar plate (Corning, Lowell, MA), at 485nm excitation and 535nm emission wavelengths. The instrument gain was set to optimize differentiation of samples in the experiment. One well of each plate contained as standard consisting of the same set of kernels to control for instrument drift during the course of measurements. When the standard values were included as a covariate in the analysis, the effect of the standard was not significant so the standard values were not used in the analyses presented here. Wells were filled with random visually positive seeds. Each plate was shaken and measured 5 times for a total of 80 individual fluorescence measurements per sample to ensure a representative measurement of the sample. Throughout the experiment, only ears that were visually determined to be segregating for GFP activity, as shown in Fig 2, were eligible for analysis.

### Quantification of seed storage proteins

To determine whether selection for GFP with the 27kDa gamma zein promoter resulted in changes in levels of the native 27kDa gamma zein, we used HPLC to quantify alcohol soluble seed storage proteins. Two samples, consisting of either random visually GFP positive or random visually GFP negative seeds, were taken from 20 random ears from each of the 4 populations in generation 3: BS11 S3, BS11 U3, BS31 S3, and BS31 U3.

Each sample was ground to fine flour and alcohol-soluble proteins were extracted with an alcohol-based buffer, as described by Flint-Garcia [16]. The proteins were separated with HPLC on a C18 protein and peptide column in a Waters 2695 Separation Module with a gradient of water and acetonitrile, both with 0.01% trifluoroacetic acid. The flow rate was 0.5ml/min and the concentration of water changed as follows: 50% at 0min, 46% at 33min, 40% at 35min, to 20% at 37min which was held for 15min. Absorbance was measured at 200nm with a Waters 2487 Dual Absorbance Detector. Individual peak areas for each sample were integrated using Empower software (Waters, Milford, MA) with a minimum peak width of 30 and threshold of 800. We grouped peaks by retention time and identified the 27kDa gamma zein and the alpha zein region by comparison to known HPLC profiles [17, 18]. A standard sample was run with each analysis batch to control for variation among batches but no significant variation in the standard values was observed so the data from the standard was not used in the analysis presented here.

### Additional phenotypic evaluation

To determine the extent of change of unselected traits in the course of the experiment, we evaluated three phenotypic traits unrelated to the transgene: germination rate, seed mass, and percent nitrogen. Germination rates for each of the 12 populations planted in year 4 were determined by counting the number of plants in each row, not including tillers. Seed mass of each of the 12 populations planted in year 4 was determined by weighing two samples of 50 randomly selected seeds from each ear that was segregating for GFP. Percent nitrogen of 0.5g of two flour samples, consisting of either visually GFP positive or visually GFP negative seeds, from 10 randomly selected ears from S3 and U3 of both breeding populations (40 samples total) was determined by combustion analysis by the Iowa State University Soil and Plant Analysis Laboratory. For all phenotypes, means for each treatment were used for statistical analysis described below.

Selection was carried out based on ears rather than seeds, with random mixtures of GFP positive and GFP negative seeds from segregating ears used to advance both selected and unselected populations each year. Each population was expected to have approximately 50% of all ears segregating for GFP, 25% with all positive seeds, and 25% with all negative seeds. To determine whether selection was affecting zygosity of the population, we determined the percentage of total harvested ears per population that were segregating for GFP. Ears with all GFP-negative seeds cannot be visually distinguished from ears with uniform low activity, so only segregating ears were counted for the purpose of determining the percentage of segregating ears in each population.

### Statistical analysis

For each of the three generations, there were two pairs of selected and unselected populations, in two breeding populations. To determine if selection was effective, we used least squares fitting of the data to a linear model. The model used for fluorescence, germination rate, and seed mass is as follows:

$${\text{Y}}_{\text{ijk}}=\mu +{\text{Gen}}_{\text{i}}+{\text{SorU}}_{\text{j}}+{\text{Pop}}_{\text{k}}+{\left(\text{Gen x SorU}\right)}_{\text{ij}}+{\left(\text{Gen x Pop}\right)}_{\text{ik}}+{\left(\text{SorU x Pop}\right)}_{\text{jk}}+{\text{error}}_{\text{ijk}}$$

Where Y is the observed value of the treatment and:

μ = the overall mean of the observed values

Gen = the effect of generations of selection (1, 2 or 3)

SorU = the effect of selection for GFP levels versus the unselected control

Pop = the effect of breeding population, BS11 or BS31

All effects were considered fixed effects, limiting the inference space to the observations made in this study. The significance of the SorU term was the test used to determine if selection was effective.

For zein peak area and total protein, we examined the variation within the most advanced generations of selection (S3 and U3). ANOVA was carried out with JMP [19], using the following fixed effects model:

$${\text{Y}}_{\text{ijk}}=\mu +{\text{GFP}}_{\text{i}}+{\text{SorU}}_{\text{j}}+{\text{Pop}}_{\text{k}}+{\text{error}}_{\text{ijk}}$$

Where Y is the observed value of the treatment, and:

μ = the overall mean of the observed values

GFP = visually positive or negative for GFP fluorescence

SorU = the effect of selected or unselected

Pop = the effect of breeding population, BS11 or BS31

A chi square test was used to determine whether the percentage of total harvested ears per population that were segregating for GFP varied significantly from the expected 50%.

## Results

### Fluorescence was increased by selection

We first set out to determine if transgene activity (measured as florescence of GFP in whole seeds) could be increased by recurrent selection. Selected and unselected populations were developed from two different starting populations, the broad-based synthetics BS11 and BS31. After three generations of breeding, we evaluated the populations for fluorescence. The data resulting from this evaluation are presented in S1 Table.

**Fig 3:**
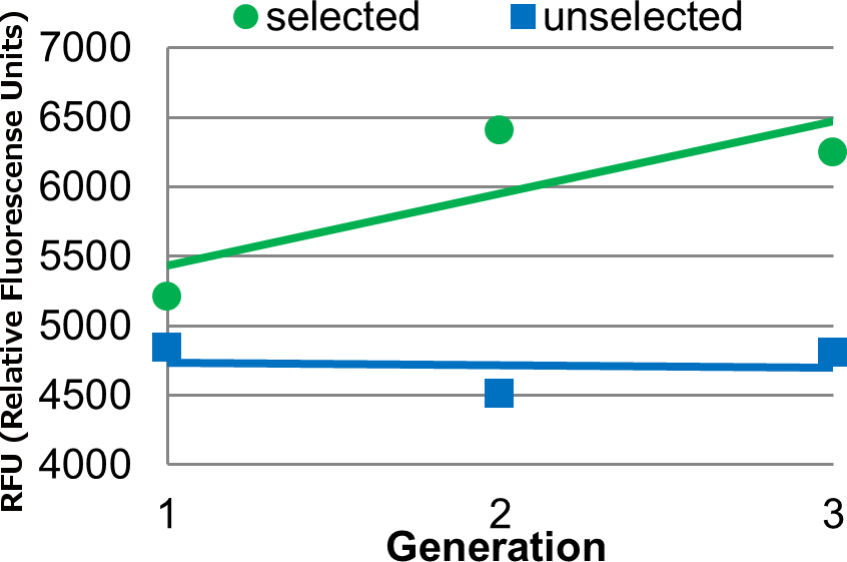
Effect of selection on seed fluorescence. Data points are mean fluorescence values versus generation, shown with the linear regression trend line. The equations of the lines for the selected and unselected populations are y = 520.43x + 4910.3 (R^2^ = 0.6397) and y = -15.242x + 4,747.8 (R^2^ = 0.0072) respectively.

The null hypothesis was that the fluorescence of the selected populations was not different than the fluorescence of the unselected populations (the SorU effect in Table 1). This effect was significant, with the fluorescence in the selected populations being 26% higher than the unselected populations (mean of selected = 5,951, mean of unselected = 4,717, standard error = 20.6; Fig 3). The significance of the SorU x Pop term in the model indicates that the difference between the selected and unselected populations was not the same in the BS11—and BS31-derived populations, however, the mean of the selected populations was higher than the mean of the unselected populations in both cases. Thus, we successfully increased grain fluorescence by selection in two different breeding populations.

**Table 1:** Analysis of variance of fluorescence, germination rate, and seed mass.

		Fluorescence		Germination rate		Seed mass	
		(relative units)		(proportion)		(grams)	
Source	DF	Sum of Squares	p value	Sum of Squares	p value	Sum of Squares	p value
Gen^a^	2	5,999,973	0.0004 **	0.2967	0.9059	1.164	0.7198
SorU^b^	1	4,567,379	0.0006 **	0.8533	0.5171	1.160	0.4711
Pop^c^	1	6,171,304	0.0004 **	0.1200	0.7975	1.058	0.4887
Gen x SorU	2	1,222,521	0.0041 **	0.7817	0.7822	2.385	0.5562
Gen x Pop	2	55,043	0.0847	1.3400	0.6768	1.934	0.6071
SorU x Pop	1	608,214	0.0042 **	0.4033	0.6455	3.763	0.2534
Model	9	13,224,435		3.7900		11.464	
Error	2	5,094		2.8067		2.988	
Total	11	13,229,529	0.0017 **	6.5967	0.9174	14.452	0.6474
R^2^		0.9996		0.5745		0.7932	

** Indicates statistical significance below the threshold of P = 0.01. Effects in the model are as follows

^a^ The effect of generations of selection.

^b^ The effect of selected vs. unselected advanced populations.

^c^ The effect of breeding population, BS11 vs BS31.

The most likely explanation for increased grain fluorescence is increased activity of GFP in the transgenic seeds. However, it is possible that increased fluorescence was somehow due to altered seed morphology, for example an increase in transparency of the pericarp or a change in seed properties that allows GFP fluorescence to be detected more readily in the selected populations. Visible examination of the seeds did not reveal any clear morphological differences between the seeds in the selected and unselected populations.

### 27kDa gamma zein was increased by selection

After finding that GFP fluorescence controlled by the 27kDa gamma zein promoter was increased by recurrent selection, we set out to determine whether levels of native 27kDa gamma zein were different in the most advanced selected and unselected populations: S3 and U3. This could happen if, for example, selection for GFP fluorescence caused an increase in transcription from the 27kDa zein promoter, or an increase in total protein content in seeds. HPLC data on zein peak areas (S2 Table) were analyzed to identify changes in zein levels in the populations of interest. The null hypothesis was that 27kDa gamma zein would not be significantly different between the selected and unselected populations. This was tested using ANOVA (Table 2). The effect of interest is SorU, which was significant, with mean 27kDa gamma zein levels (measured as peak area) being 22.73% higher in selected samples compared to unselected control samples (mean of selected = 8,638,293 and unselected = 7,594,531, standard error = 235,673). Notably, 27kDa gamma zein levels were not significantly different between GFP positive and negative samples in either breeding populations. This shows that selection for GFP controlled by the 27kDa gamma zein promoter did increase 27kDa gamma zein levels but that presence of the transgene had no effect. Population had a significant effect on 27kDa gamma zein levels, as was expected because zein levels are known to vary across breeding populations.

**Table 2:** Analysis of variance of 27kDa gamma zein peak area and percent nitrogen.

		27kDa zein		Percent nitrogen	
		(relative area)		(proportion)	
Source	DF	Sum of Squares	p value	Sum of Squares	p value
GFP^a^	1	14.63	0.4626	0.0181	0.0829
SorU^b^	1	498.55	0.0091 **	0.0422	0.0247 **
Pop^c^	1	675.75	0.0053**	0.1170	0.0043 **
Model	3	1,188.93		0.1773	
Error	4	88.87		0.0137	
Total	7	1,277.79	0.0089 **	0.1911	0.0094 **
R^2^		0.8345		0.9282	

** and * indicate statistical significance below the thresholds of P = 0.01 and P = 0.05, respectively. Effects in the model are as follows

^a^ The effect of visually GFP positive vs. visually negative seeds.

^b^ The effect of selected vs. unselected populations.

^c^ The effect of breeding population, BS11 vs BS31.

**Fig 4:**
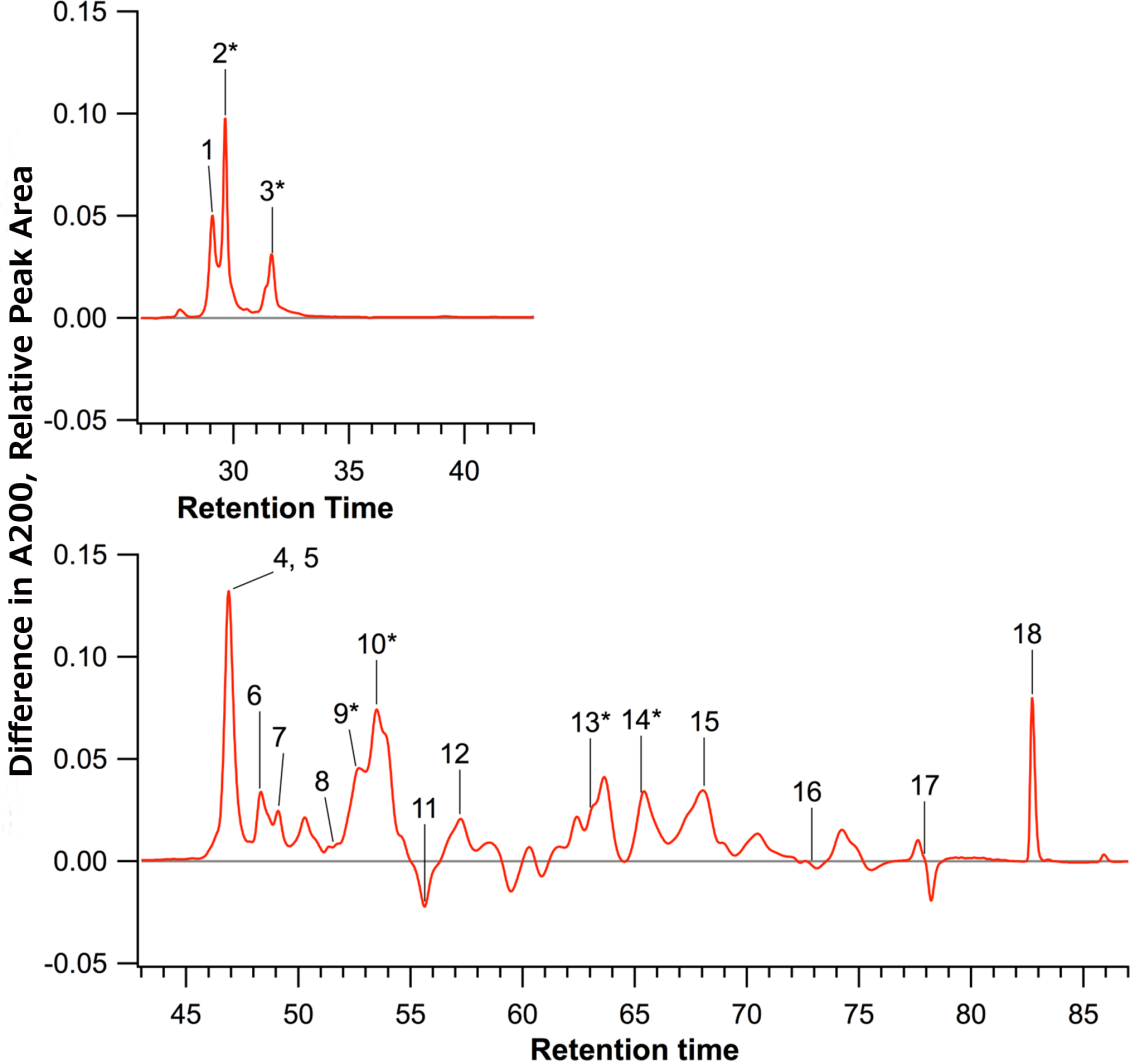
Composite HPLC traces. The red line is the mean of traces of the selected population minus the mean of the traces of the unselected population. Peak 2 is the 27kDa gamma zein, identified by comparison to known HPLC traces [17, 18]. Asterisks indicate peaks that were significantly higher in the selected samples (α ≤ 0.05).

In addition to the 27kDa gamma zein, 5 of the 17 other zeins were significantly increased in the selected samples compared to the unselected samples (peaks 2, 3, 9, 10, 13, and 14), and no zeins were significantly decreased. Breeding population had a significant effect on 4 zeins (peaks 1, 8, 14, and 16). ANOVA tables for all zeins are in S3 Table. We created a composite HPLC trace by subtracting the mean absorbance values from the unselected control populations from those of the selected populations (Fig 4).

It appears that selection for fluorescence increased levels of certain seed storage proteins, most notably the 27kDa gamma zein, which has the same promoter as the GFP transgene construct. It is therefore possible that selection for fluorescence resulted in up-regulation of zein production by up-regulation of zein promoters, however it is also possible that the observed increase in fluorescence and seed storage protein levels result from other changes such as an increase of total protein concentration or some other more complex trait. We next examined other traits to see if this was the case.

### Selection for fluorescence caused minimal changes in other traits

There were no overall visual differences between the populations but plants within each population did vary visually for height, color, and other characteristics, as is expected for segregating populations. We quantified three traits unrelated to fluorescence to determine whether there were significant differences between selected and unselected populations: germination rate, seed mass, and percent nitrogen (S3 Table). For germination rate and seed mass, significance of the effect SorU would indicate there was a difference between the selected and unselected populations. For percent nitrogen, significance of the SorU effect would indicate a significant difference between the selected and unselected populations in the third generation.

**Fig 5:**
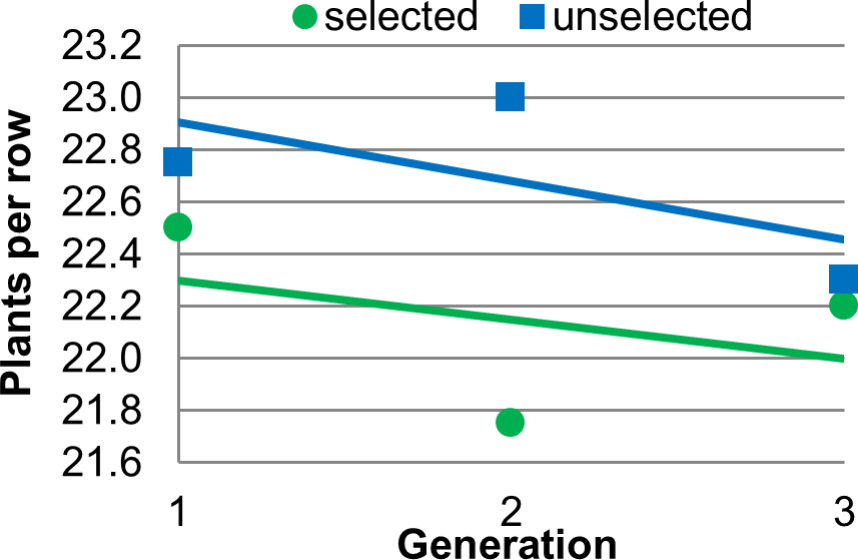
Effect of selection on germination rate. Data points are mean number of plants per row values versus generation, shown with the linear regression trend lines. The equations of the lines for the selected and unselected populations are y = -0.15x + 22.45 (R^2^ = 0.1579) and y = -0.225x + 23.133 (R^2^ = 0.4023) respectively.

The SorU effect was not significant for germination rate (Fig 5), nor was any other model effect, indicating that this trait was not altered by selection. The difference between the means of the selected and unselected populations was only 0.4 plants per plot (unselected = 22.6 and selected = 22.2, standard error = 0.48). The ANOVA table for germination rate is in Table 1.

**Fig 6:**
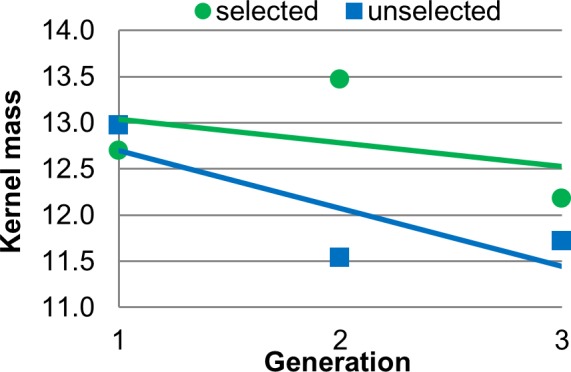
Effect of selection on kernel mass. Data points are mean 50-seed mass (grams) versus generation, shown with the linear regression trend lines. The equations of the lines for selected and unselected populations are y = -0.2559x + 13.294 (R^2^ = 0.1554) and y = -0.6242x + 13.325 (R^2^ = 0.6387) respectively.

Seed mass was similar to germination rate in that the SorU effect was not significant (Table 1, Fig 6), leading us to conclude there was no effect of selection. The difference between the selected and unselected populations was only 0.6 grams per 50 kernels (unselected = 12.16 and selected = 12.78, standard error = 0.50).

Percent nitrogen was measured only in the populations from the third generation (similar to the zein analysis) and was 11% higher in selected populations compared to unselected populations (n = 40 samples made up of 2 samples each from 10 ears per population). The mean percent nitrogen was 1.42 for ears in the unselected populations and 1.57 for ears in the selected populations. Thus, an increase in total protein content due to selection can partly explain the differences in GFP fluorescence and zein content, but the size of the change is not sufficient to explain either change completely. In addition, there was no significant difference in percent nitrogen between visually GFP positive and negative seeds, indicating that the transgene did not have an effect on total protein. The ANOVA table for percent nitrogen is in Table 2.

**Fig 7:**
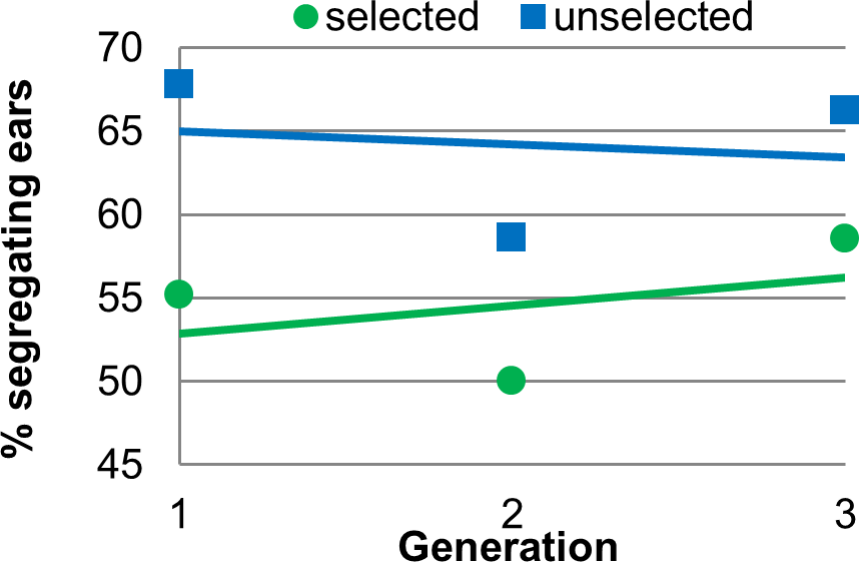
Effect of selection on percentage of segregating ears. Data points are percentage of segregating ears versus generation, shown with linear regression trend lines. The equations of the lines for selected and unselected populations are y = 1.6691x + 51.223 (R^2^ = 0.1515) and y = -0.7808x + 65.75 (R^2^ = 0.0251) respectively. There is significant deviation from the expected 50% segregating ears in the unselected populations, but not in the selected populations (α ≤ 0.05).

There was significant deviation from the expected 50% segregating ears in the unselected populations (64.53%, p = 0.0050, n = 234 ears) but not in the selected populations (55.09%, p = 0.4334, n = 216 ears). This indicates that zygosity was not changed with selection for increased fluorescence. While the unselected populations had more segregating ears than expected, there is no trend over generations, as shown in Fig 7.

## Discussion

The objective of this research was to determine if transgene activity could be changed by recurrent selection and we found that it was. These findings provide a new way to increase levels of transgene activity. We also determined the effects of selection for transgene activity on an endogenous gene with the same promoter as the transgene, finding that the protein produced by the endogenous gene was increased.

Fluorescence of GFP controlled by the 27kDa gamma zein promoter was significantly increased with three generations of selection in two breeding populations. Even though the native 27kDa gamma zein was not the target of selection, its protein level was significantly increased in the selected populations of both breeding populations. The magnitudes of the increase in fluorescence and 27kDa gamma zein levels in generation 3 are similar. For fluorescence, the selected populations were 17.28 and 48.58% higher than the unselected populations for BS11 and BS31 respectively. For 27kDa gamma zein levels, the selected populations were 14.35 and 31.40% higher than the unselected populations for BS11 and BS31 respectively. We hypothesize that selection acted on regulatory sequences common to the transgene and the native 27kDa gamma zein gene. Since the common element between the transgene and the native gene is the promoter, it seems likely that selection had an impact on transcription, possibly through altered activity of one or more transcription factors. Additional studies, such as RNASeq for known 27kDa gamma zein transcription factors, are needed to determine if this is the case. It is important to note that the 3’ untranslated region of the transgene was not derived from a zein gene. Apparently, sufficient regulatory information resides in the 5’ untranslated region to allow selection for the transgene to impact levels of the zein. It would be interesting to repeat the experiment using the native 3’ untranslated region of the gene as well.

In addition to significant increases in 27kDa gamma zein levels, multiple other zeins also had significant increases in the selected populations. These increases may be caused by transcription factors that are shared between the 27kDa gamma zein gene and those zein genes, such as PBF-1, which was shown by Wang et al. to bind to the 27, 22, and 19kDa zein promoters [20]. The 27kDa gamma zein plays a role in protein body formation, and stabilizes other zeins [21], so increased level of the 27kDa gamma zein in the selected populations may be contributing to higher stability of other zeins. This effect has been seen in quality protein maize, where higher level of 27kDa gamma zein is associated with seed vitreousness [22]. Alternatively, the significant differences in zeins could be due to genetic drift or to genotypic differences. For example, gamma zein level is highly variable across genotypes [16]. However, the lack of significant differences between selected and unselected populations for germination rate and seed mass indicates that genetic drift is not occurring for these traits, or that it is occurring at the same rate and in the same direction in the selected and unselected populations.

Notably, there were no significant differences in 27kDa gamma zein level between GFP positive and negative seeds on ears segregating for the transgene. GFP negative seeds in the selected populations had elevated levels of 27kDa gamma zein that were just as high as levels in GFP positive seeds, indicating that genetic changes resulting from selection are not dependent on the presence of the transgene. This change in protein level of one gene through selection of another gene could be thought of as selection by proxy, or proxy selection. Proxy selection could be a way to use a reporter transgene as a breeding tool to alter the expression of a native gene that shares regulatory elements with the reporter transgene. The transgene can be segregated out after selection, leaving no transgene in the final product.

In this study, the level of the native 27kDa gamma zein gene was increased by recurrent selection for activity of a GFP transgene with the 27kDa gamma zein promoter. Proxy selection has the potential to be a useful tool to alter expression of native genes whose products are difficult to quantify.

## Supporting Information

S1 Table Plot means for Fluorescence, Germination Rate, Seed mass, 27 kDa gamma zein and Nitrogen. (XLSX)

S2 Table Zein peak information. See Fig 4 for peak assignments.
(XLSX)

S3 Table ANOVAs of zein peak areas. See Fig 4 for peak assignments.
(XLSX)
